# A Head-to-Head Comparison of Two Antibodies for HER2 Pretargeted PET Imaging via Bioorthogonal Click Chemistry

**DOI:** 10.3390/ph19081276

**Published:** 2026-08-13

**Authors:** Yong Huang, Chengze Li, Taichuang Li, Jiuhui Zhao, Xinyu Yang, Maoqun Zhang, Ying Liang

**Affiliations:** Department of Nuclear Medicine, National Cancer Center/National Clinical Research Center for Cancer/Cancer Hospital & Shenzhen Hospital, Chinese Academy of Medical Sciences and Peking Union Medical College, Shenzhen 518116, China; 13260455651@163.com (Y.H.); lichengze1915@163.com (C.L.); 17688426991@139.com (T.L.); izljkh@163.com (J.Z.); 13875015513@163.com (X.Y.); libseveny@163.com (M.Z.)

**Keywords:** pretargeted PET imaging, HER2, Diels-Alder reaction, Pertuzumab-TCO/[^18^F]PEG_12_-Tz

## Abstract

**Background/Objectives:** Antibody selection is critical for advancing pretargeted PET imaging toward clinical translation. This study directly compared two clinically approved anti-HER2 antibodies—trastuzumab and pertuzumab—within an identical pretargeting system, utilizing the specific chemical ligation between tetrazine and trans-cyclooctene (TCO) via the inverse electron-demand Diels–Alder (IEDDA) cycloaddition. **Methods:** In HER2-positive SKOV3 tumor-bearing mice, TCO-conjugated antibodies were administered 1–13 days prior to injection of a fluorine-18-labeled tetrazine probe ([^18^F]PEG_12_-Tz). **Results:** Both strategies enabled high-contrast imaging, overcoming the kinetic mismatch between slow-clearing antibodies and the short-lived ^18^F isotope. Distinct profiles were observed: pertuzumab-TCO yielded higher tumor uptake and a broader imaging window (3–13 days), whereas trastuzumab-TCO resulted in lower uptake and a narrower window (1–5 days). These differences are not affinity-driven, as both conjugates showed similar Kd values (~11–14 nM). Instead, pertuzumab-TCO’s superiority stems from a dual mechanism: (1) slower cellular internalization (65.1% vs. 94.0% at 60 min) preserves surface TCO for click reaction; and (2) markedly slower blood clearance (t_1/2β_ = 285.9 h vs. 106.0 h) ensures sustained bioavailability and tumor extravasation. **Conclusions:** This head-to-head evaluation demonstrates that epitope-dictated internalization and clearance are decisive parameters for antibody selection, providing a mechanism-based framework for optimizing pretargeted imaging.

## 1. Introduction

In oncology, accurate detection and quantification of human epidermal growth factor receptor 2 (HER2) expression are crucial for patient stratification, therapeutic decision-making, and treatment response assessment [1,2]. Immuno-positron emission tomography (immuno-PET), which employs radiolabeled monoclonal antibodies for molecular imaging, provides a promising non-invasive method for in vivo visualization of HER2 expression [3,4,5,6,7,8,9,10,11,12]. However, a major limitation stems from the mismatch between the pharmacokinetics of antibodies and the physical half-lives of clinically applicable radionuclides [5]. Monoclonal antibodies exhibit slow systemic clearance and extended circulation times, necessitating the use of long-half-life radionuclides such as zirconium-89 (t_1/2_ ≈ 78.4 h) to achieve sufficient tumor-to-background contrast [13]. Consequently, imaging is typically delayed to 4–7 days post-administration, leading to increased radiation burden and logistical challenges [14]. Coupled with the limited availability of long-half-life isotopes, these constraints hinder the broad clinical adoption and serial application of conventional immuno-PET [15,16,17,18].

Pretargeting strategies have emerged as an effective approach by decoupling the slow tumor-targeting phase of antibodies from the rapid imaging kinetics of short-half-life radionuclides [19,20]. In this method, a non-radioactive, tumor-targeting antibody conjugate is first administered, allowing sufficient time for tumor accumulation and systemic clearance. Subsequently, a small, rapidly clearing radioligand with high affinity for the pre-localized antibody is injected. Among available pretargeting methodologies, bioorthogonal chemistry—particularly the inverse electron-demand Diels-Alder (IEDDA) reaction between tetrazine (Tz) and trans-cyclooctene (TCO)—has garnered considerable attention due to its rapid reaction kinetics, high selectivity, and excellent biocompatibility. This approach enables the use of short-lived, clinically favorable PET radionuclides such as fluorine-18 (t_1/2_ ≈ 109.8 min), thereby facilitating same-day imaging with high contrast and reduced radiation exposure [21,22,23,24,25,26,27,28,29,30,31].

Trastuzumab and pertuzumab are two cornerstone monoclonal antibody therapies targeting HER2, binding to distinct extracellular subdomains (Domain IV and Domain II, respectively) [32,33,34]. While their therapeutic synergy is well established, their comparative performance as targeting components within a bioorthogonal pretargeted PET framework remains uncharacterized. This knowledge gap is critical, as antibody selection can profoundly influence pretargeting efficiency through factors such as binding affinity, internalization dynamics, and in vivo pharmacokinetics [35,36]. Therefore, a systematic head-to-head evaluation is necessary to inform the rational design of optimized pretargeted imaging protocols.

Here, we employed a unified bioorthogonal pretargeting platform based on the Tz-TCO IEDDA reaction, as previously reported [31], to directly compare trastuzumab and pertuzumab. TCO-conjugated trastuzumab or pertuzumab were used as targeting vectors, in conjunction with a fluorine-18-labeled tetrazine tracer ([^18^F]PEG_12_-Tz), to establish two pretargeted imaging strategies. This study aims to elucidate how the distinct molecular characteristics of these clinically relevant antibodies affect tumor targeting, pharmacokinetics, and overall imaging performance in HER2-positive xenograft models. The findings provide essential insights for guiding antibody selection and optimization, thereby advancing the clinical translation of pretargeted PET imaging.

## 2. Results

### 2.1. Chemical and Radiochemical

The TCO-functionalized trastuzumab/pertuzumab conjugates were successfully engineered by incubating the native antibodies with a roughly 40-fold molar excess of NHS-PEG4-TCO at room temperature for 1 h. Following conjugation, the recovery yield of the antibody conjugates was 83.3 ± 6.0%, and subsequent HPLC purification ensured a final product purity exceeding 95%. The mass spectra of trastuzumab (found 148.22 kDa), pertuzumab (found 148.48 kDa), trastuzumab-TCO (found 153.40 kDa) and pertuzumab-TCO (found 160.68 kDa) are shown in Figures S1–S4. Furthermore, MALDI-TOF-MS characterization revealed an average TCO loading of approximately 13 modifications per trastuzumab molecule and 30 modifications per pertuzumab molecule. Size-exclusion HPLC (SEC-HPLC) confirmed that both conjugates were >95% monomeric, with <5% aggregation (Figures S5 and S6).

**Figure 1:**
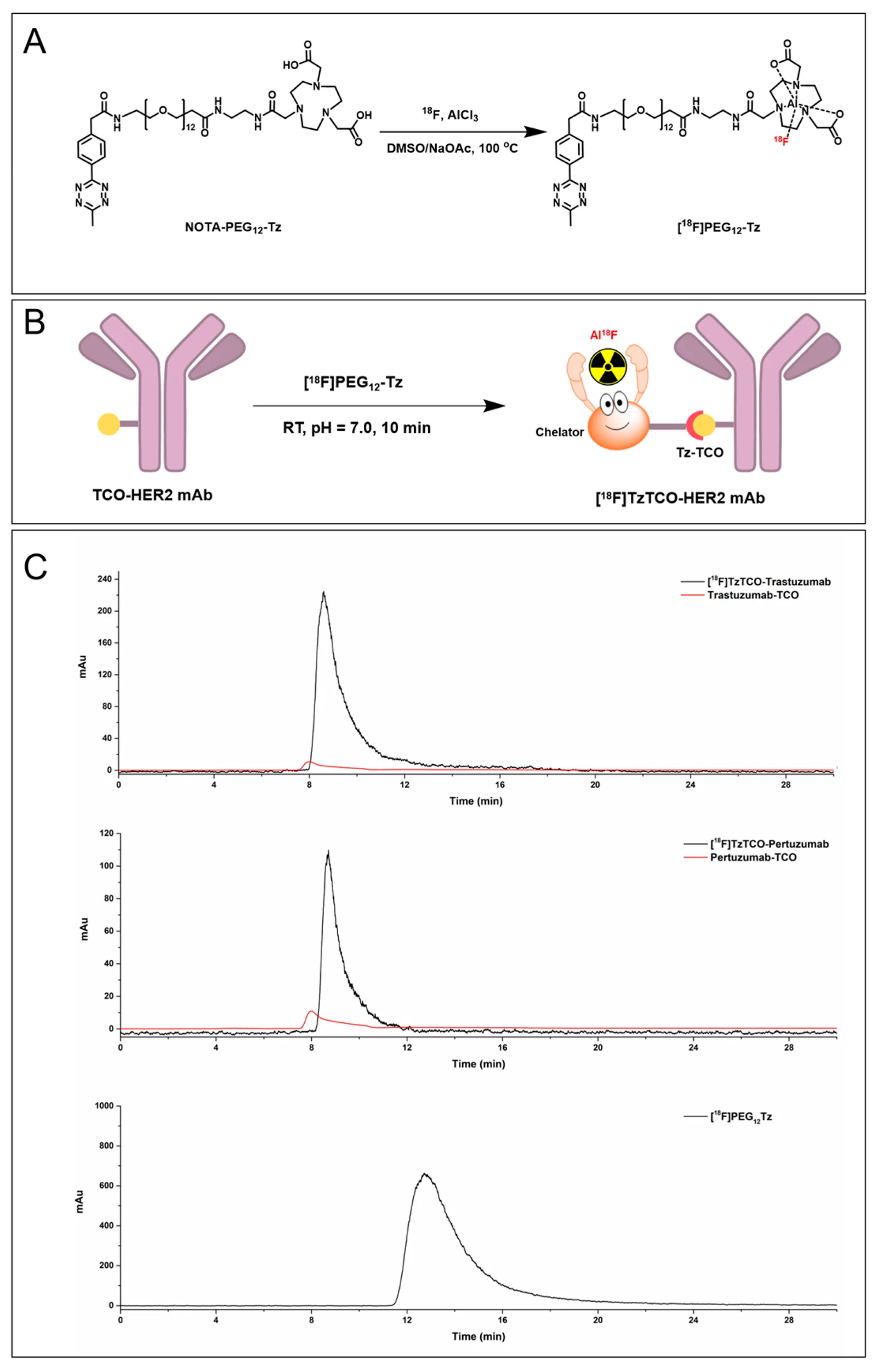
(**A**) Radionuclide labeling of NOTA-PEG_12_-Tz with fluorine-18; (**B**) Schematic of the bioorthogonal ligation between TCO-functionalized HER2 monoclonal antibodies and [^18^F]PEG_12_-Tz; (**C**) The quality control radioactive chromatography of [^18^F]TzTCO-HER2 mAb-TCO, displaying both the radioactivity (black line) and UV absorption (red line) profiles.

[^18^F]PEG_12_-Tz was radiolabeled by complexing Al^18^F with a NOTA chelator and was successfully synthesized via a one-step manual labeling procedure (Figure 1A). The radiosynthesis utilized 25–35 GBq of starting [^18^F]fluoride and 200 nmol of the NOTA-PEG_12_-Tz precursor, yielding 7.2–10.5 GBq of isolated product (non-decay-corrected yield: 28.9% ± 5.0%; decay-corrected yield: 52.3% ± 6.8%, n = 20). The final formulation was prepared in 10% ethanol/saline (pH 6.8–7.2), with residual ethanol content below 0.5% (*v*/*v*). Quality control results showed a radiochemical purity of >98% (retention time = 9.13 min; see Supplementary Figure S7), and structural identity was confirmed by co-injection with a cold standard (retention time = 8.65 min; Figure S7). The LogD value was measured as −3.03 ± 0.25, and the molar activity was 3.2 ± 0.4 GBq/μmol. The total radiolabeling time, including labeling and purification, was approximately 20 min. Trastuzumab/pertuzumab-TCO were indirectly radiolabeled with [^18^F]PEG_12_-Tz, yielding a radiochemical efficiency of 38.15 ± 4.2% (n = 5) and a molar activity of 55.5 ± 5.3 GBq/μmol (Figure 1B). [^18^F]TzTCO-Trastuzumab/Pertuzumab showed purities of >95% (retention times: 8.57 min and 8.85 min, respectively), with no detectable free [^18^F]PEG_12_-Tz (retention time: 12.6 min; Figure 1C).

### 2.2. In Vitro Stability

Following a 7-day storage period in PBS at 4 °C, SEC-HPLC analysis indicated that the pertuzumab-TCO conjugate retained a monomeric content of >95% (Figure S8), demonstrating remarkable storage stability. Additionally, serum stability assays (37 °C, 1 h) showed that both [^18^F]PEG_12_-Tz and [^18^F]TzTCO-Pertuzumab maintained radiochemical purities exceeding 95% (Figures S9 and S10), verifying their robust stability under physiological conditions.

### 2.3. Cell Assay

Western blot analysis demonstrated that HER2 was markedly overexpressed in the SKOV3 cell line, whereas its expression was minimal in the MDA-MB-231 cell line (Figure S11). Quantitative assessment of the HER2/GAPDH ratio yielded values of 5.01 ± 0.26 in SKOV3 cells and 0.024 ± 0.003 in MDA-MB-231 cells, confirming significantly higher HER2 expression in SKOV3 cells. Based on these results, SKOV3 cells were selected for subsequent in vitro experiments evaluating pre-targeting specificity, cellular uptake, internalization, efflux, and saturation binding.

**Figure 2:**
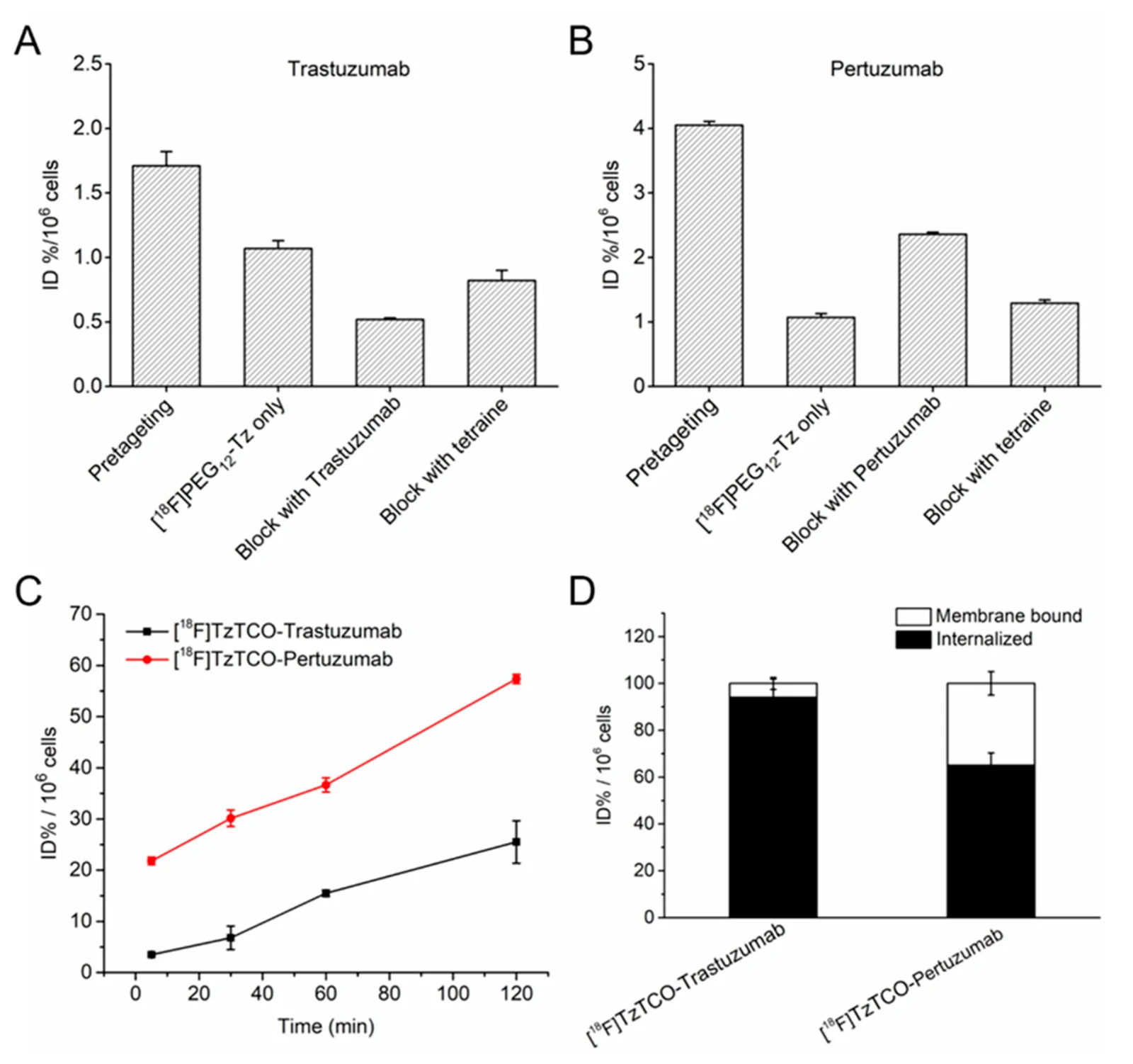
Cellular binding of [^18^F]PEG_12_-Tz to SKOV3 cells under various blocking conditions. Cells were pre-incubated with trastuzumab-TCO (**A**) or pertuzumab-TCO (**B**) for 1 h prior to radioligand addition (first bar); [^18^F]PEG_12_-Tz was added directly to cells without pre-incubation (second bar); cells were co-incubated with an excess of unlabeled trastuzumab/pertuzumab alongside the TCO-conjugates for 60 min before radioligand challenge (third bar); and cells pre-treated with the TCO-antibodies were subsequently exposed to an excess of unlabeled tetrazine prior to [^18^F]PEG_12_-Tz addition (fourth bar). (**C**) Cellular uptake and (**D**) internalization profiles of [^18^F]TzTCO-Trastuzumab and [^18^F]TzTCO-Pertuzumab in SKOV3 cells. Data are presented as means ± SD (%ID/g) (n = 3).

In vitro binding of [^18^F]PEG_12_-Tz to trastuzumab/pertuzumab-TCO pretreated SKOV3 (HER2-positive) cells was 1.61- and 3.79-fold higher than nonspecific uptake in untreated cells, respectively (Figure 2A,B). Moreover, [^18^F]PEG_12_-Tz binding to SKOV3 cells was substantially reduced when trastuzumab/pertuzumab-TCO binding was blocked by receptor pre-saturation with unmodified anti-HER2 antibodies or by competition with a large excess of unlabeled tetrazine (Figure 2A,B). Cellular uptake studies of [^18^F]TzTCO-Trastuzumab and [^18^F]TzTCO-Pertuzumab were performed to assess the binding capacity of the two antibodies to HER2. As shown in Figure 2C, accumulation of [^18^F]TzTCO-Trastuzumab and [^18^F]TzTCO-Pertuzumab in SKOV3 cells reached 3.50 and 21.82 ID%/10^6^ cells at 30 min, respectively. With extended incubation up to 120 min, uptake of both agents increased, with [^18^F]TzTCO-Pertuzumab exhibiting a greater time-dependent increase compared to [^18^F]TzTCO-Trastuzumab. At 120 min, their respective uptakes reached 25.51 and 57.36 ID%/10^6^ cells. Internalization assays indicated that both trastuzumab and pertuzumab underwent endocytosis. Notably, [^18^F]TzTCO-Trastuzumab showed a significantly higher internalization rate of 94.0% in SKOV3 cells compared to 65.1% for [^18^F]TzTCO-Pertuzumab at 60 min (Figure 2D). Efflux experiments revealed moderate cellular efflux for [^18^F]TzTCO-Trastuzumab and more rapid efflux for [^18^F]TzTCO-Pertuzumab. At 180 min, [^18^F]TzTCO-Trastuzumab retained 69.6% of its initially accumulated radioactivity, while [^18^F]TzTCO-Pertuzumab retained only 29.9% (Figure S12). Saturation binding assays revealed stable nonspecific (NS) binding for both [^18^F]TzTCO-Trastuzumab and [^18^F]TzTCO-Pertuzumab (Figure S13). In parallel, the specific binding (SB) isotherms displayed typical sigmoidal profiles, with equilibrium dissociation constants (Kd) calculated as 11.46 nM and 14.29 nM, respectively.

### 2.4. Micro-PET/CT Imaging, Biodistribution and Immunohistochemistry Analysis

**Figure 3:**
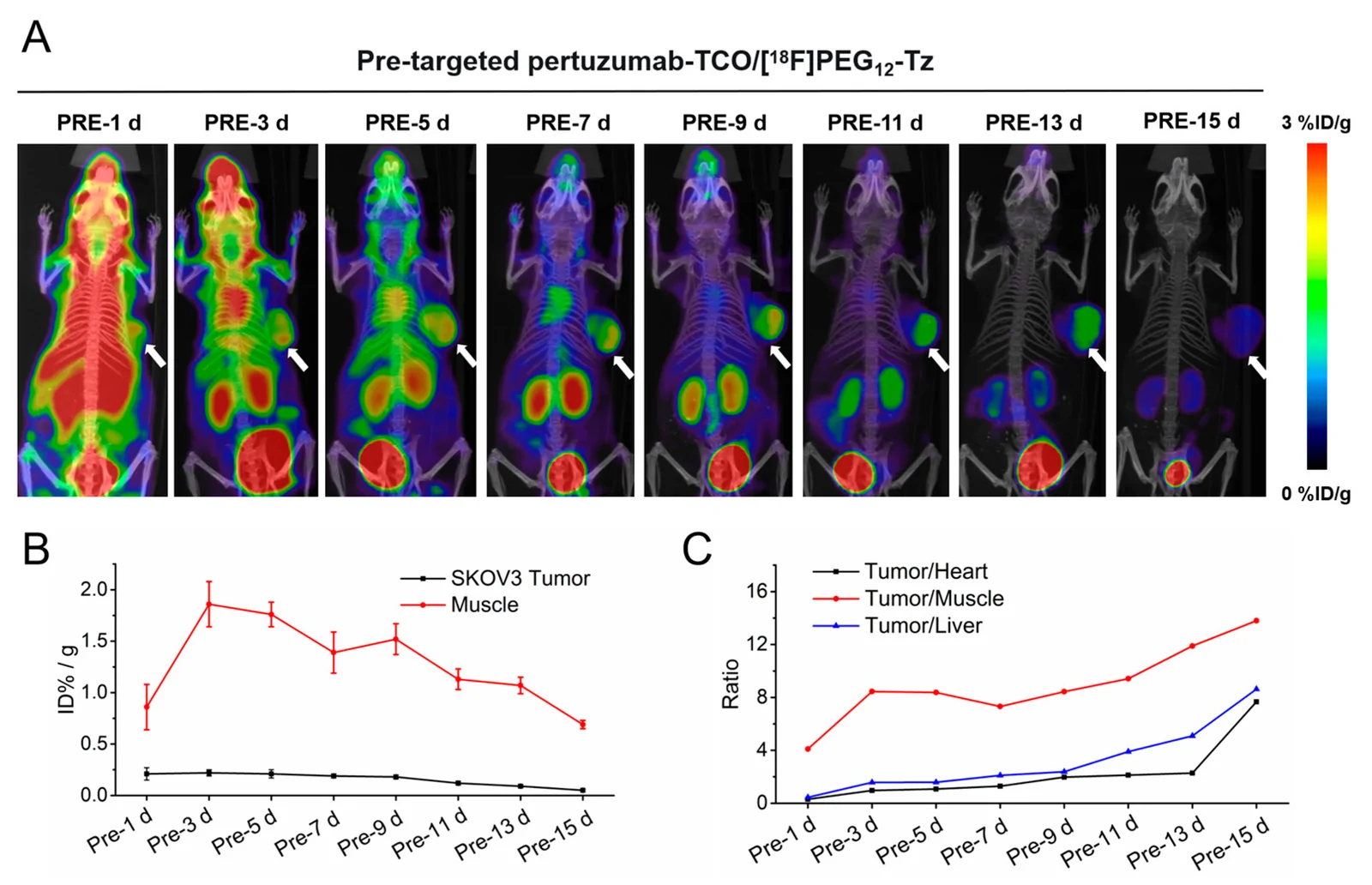
(**A**) Pretargeted micro-PET imaging of SKOV3 tumor-bearing mice following intravenous administration of pertuzumab-TCO (300 μg in PBS) and subsequent injection of [^18^F]PEG_12_-Tz at designated intervals (1, 3, 5, 7, 9, 11, 13, and 15 d later), with white arrows delineating the tumor regions. (**B**) Corresponding uptake value of SKOV3 tumors and muscles at 1 h post-administration of the radioligand. (**C**) The ratios of SKOV3 tumor/heart, tumor/muscle and tumor/liver. Error bars are defined as mean ± standard deviation (SD) (n = 3).

Figure 3A–C and Figure S14A–C show the PET imaging results of the two antibody-based pretargeting strategies. Pretargeted imaging data following administration of trastuzumab/pertuzumab-TCO indicate that both approaches effectively detect HER2 expression in vivo. Imaging performed 1 h after [^18^F]PEG_12_-Tz injection revealed peak tumor radiotracer uptake at a 3-day interval between trastuzumab/pertuzumab-TCO and [^18^F]PEG_12_-Tz administration, with uptake values of 1.42 ± 0.16% ID/g and 1.86 ± 0.22% ID/g for the trastuzumab- and pertuzumab-based pretargeting strategies, respectively, followed by a gradual decline. Meanwhile, tumor/muscle, tumor/heart, and tumor/liver ratios for both strategies progressively increased over time. A notable difference in tumor uptake and retention was observed between the two approaches. Compared with the trastuzumab-based strategy, the pertuzumab-based approach yielded higher intratumoral radiotracer accumulation and allowed for a prolonged imaging window. Consistent application of this analysis to both antibodies revealed distinct pharmacokinetic profiles. For trastuzumab-TCO, tumor uptake exceeded 1.0% ID/g on day 1 (1.30 ± 0.14% ID/g) but fell below the 1.0% ID/g threshold by day 7 (0.68 ± 0.10% ID/g); its tumor-to-muscle ratio (TMR) reached the ≥5.0 criterion during days 1–5, defining the optimal imaging window as days 1–5. In contrast, pertuzumab-TCO exhibited a delayed peak on day 3, exceeding 1.0% ID/g (1.86 ± 0.22% ID/g), which was sustained at 1.07 ± 0.08% ID/g through day 13 with a TMR consistently ≥5.0 from days 3–13. Consequently, the optimal dosing window for pertuzumab-TCO is substantially longer, spanning days 3–13.

**Figure 4:**
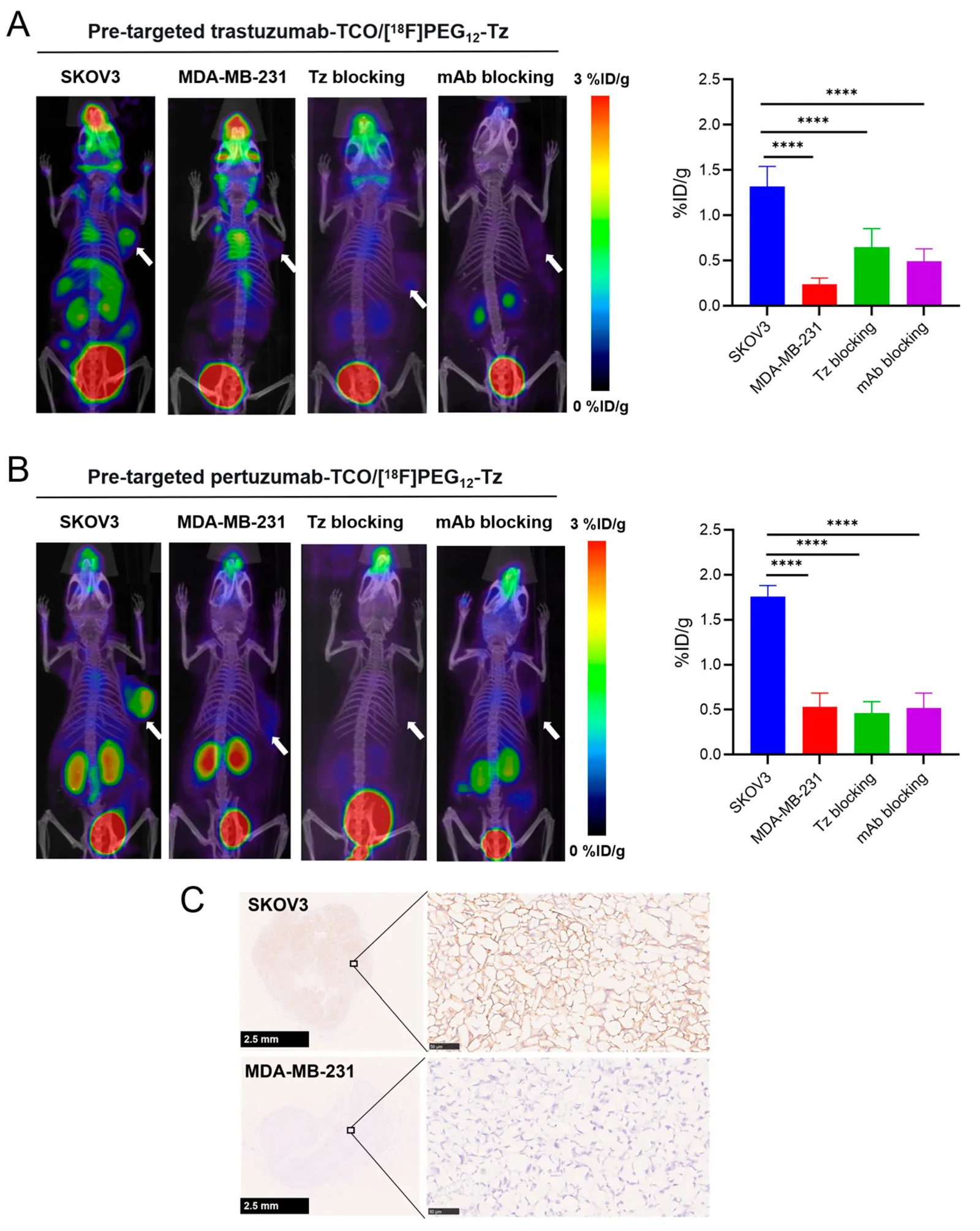
(**A**) Pre-targeted trastuzumab-TCO/[^18^F]PEG_12_-Tz images of SKOV3 tumor uptake ((**A**), left), MDA-MB-231 tumor uptake ((**A**), middle), SKOV3 tumor block with excessive Tz ((**A**), right) and SKOV3 tumor block with excessive trastuzumab. (**B**) Pre-targeted pertuzumab-TCO/[^18^F]PEG_12_-Tz images of SKOV3 tumor uptake ((**B**), left), MDA-MB-231 tumor uptake ((**B**), middle), SKOV3 tumor block with excessive Tz ((**B**), right) and SKOV3 tumor block with excessive pertuzumab. (**C**) IHC staining for HER2 expression revealing intense immunoreactivity (claybank) in SKOV3 tumors, in contrast to weak immunoreactivity observed in MDA-MB-231 tumors. Statistical significance was indicated as **** *p* < 0.001. Error bars were defined as mean ± standard deviation (SD) (n = 3).

In vivo pretargeted PET imaging and biodistribution analyses of mice harboring subcutaneous MDA-MB-231 tumors (characterized by low HER2 expression) revealed markedly diminished radioligand uptake within the tumor, failing to produce discernible contrast against adjacent tissues (Figure 4, Tables S1 and S2). Statistical analysis confirmed that tracer uptake in MDA-MB-231 tumors was markedly lower than that in SKOV3 tumors at the same pretargeting interval. In two additional blocking groups of SKOV3 tumor-bearing mice, administration of either unmodified trastuzumab/pertuzumab before the tetrazine radioligand or co-injection of unlabeled tetrazine also resulted in significantly reduced radiotracer accumulation within SKOV3 tumors. Immunohistochemical analysis of SKOV3 and MDA-MB-231 tumor sections is shown in Figure 4C. SKOV3 tumors exhibited high HER2 expression in both the cytoplasm and cell membrane, whereas MDA-MB-231 tumors showed low HER2 expression, consistent with the PET imaging results.

Biodistribution and pharmacokinetic analyses in normal female BALB/cJGpt mice revealed a significant disparity in the serum clearance kinetics of the two conjugates: pertuzumab-TCO exhibited a markedly prolonged terminal half-life (t_1/2β_ = 285.9 h), whereas trastuzumab-TCO was cleared much more rapidly (t_1/2β_ = 106.0 h) (Tables S2 and S3, Figures S17 and S18). These divergent clearance profiles ultimately dictated the differential in vivo biodistribution and systemic exposure patterns.

A comparison between pretargeted trastuzumab/pertuzumab-TCO/[^18^F]PEG_12_-Tz imaging and direct PET imaging at 1, 4, and 8 h post-injection of [^18^F]TzTCO-trastuzumab/pertuzumab was conducted using HER2-positive SKOV3 xenografts (Figure S15). Direct radiolabeling of trastuzumab and pertuzumab resulted in prominent early hepatic accumulation and slow tumor uptake. At 8 h, tumor uptake values were 0.41 ± 0.13 and 1.02 ± 0.23% ID/g, respectively, with muscle uptake of 0.13 ± 0.03 and 0.37 ± 0.08% ID/g, and liver uptake of 37.76 ± 5.23 and 38.97 ± 7.21% ID/g. The tumor-to-muscle and tumor-to-liver ratios obtained with directly labeled antibodies at 8 h were significantly lower than those achieved with the corresponding pretargeting strategies (Figure S16). These findings highlight the limitation of direct antibody imaging using short-half-life radionuclides due to mismatched pharmacokinetics, whereas the pretargeting approach effectively overcomes the slow in vivo clearance of antibodies, thereby enabling targeted imaging with short-half-life radionuclides such as fluorine-18.

## 3. Discussion

This study presents a direct, platform-controlled comparison of two clinically relevant anti-HER2 antibodies within a bioorthogonal pretargeted PET imaging framework. The principal finding is that although both trastuzumab and pertuzumab enable high-contrast imaging of HER2-positive tumors, they exhibit fundamentally distinct in vivo performance characteristics. Specifically, the pertuzumab-based pretargeting system showed greater absolute tumor uptake, prolonged intratumoral retention, and a significantly broader optimal imaging window (3–13 days) compared with the trastuzumab-based system (1–5 days). This head-to-head evaluation extends beyond demonstrating feasibility and provides a critical mechanistic insight: antibodies targeting the same antigen are not functionally equivalent in a multistep pretargeting context.

The two conjugates exhibit similar equilibrium dissociation constants (Kd: 11.46 nM for trastuzumab-TCO vs. 14.29 nM for pertuzumab-TCO), indicating that differences in HER2 binding affinity have a minimal impact on the outcomes of in vivo pretargeted imaging. Therefore, the observed divergent in vivo performances are primarily attributed to their distinct molecular interaction mechanisms with HER2 and systemic pharmacokinetic properties. Specifically, pertuzumab binds to Subdomain II of HER2, predominantly inhibiting receptor dimerization [32,33], which corresponds to a significantly slower internalization rate (65.1% at 60 min) compared to trastuzumab. Trastuzumab binds to Subdomain IV and strongly induces internalization (94.0% at 60 min, Figure 2D). The rapid internalization of trastuzumab-TCO likely sequesters a considerable fraction of conjugates within cells before radioligand administration, effectively reducing the accessible TCO density on the cell surface and resulting in a narrower imaging window. Conversely, the slower internalization of pertuzumab-TCO allows TCO moieties to persist on the tumor cell surface for an extended duration, thereby maximizing their accessibility for the subsequent click reaction.

Beyond these cellular kinetics, the differential blood clearance rates of the antibodies further dictate their in vivo imaging performance. Pharmacokinetic data (Figures S17 and S18) demonstrate that pertuzumab-TCO exhibits a markedly slower blood clearance, with a terminal half-life (t_1/2β_) of 285.9 h compared to just 106.0 h for trastuzumab-TCO. This extended systemic circulation ensures the sustained bioavailability of the antibody conjugate, facilitating its continuous extravasation and accumulation at the tumor site. Consequently, this dual mechanism—combining slower internalization with prolonged systemic exposure—underpins the significantly broader in vivo imaging window observed for pertuzumab-TCO (3–13 days) relative to trastuzumab-TCO (1–5 days). Thus, in antibody selection for pretargeted imaging, internalization kinetics and systemic clearance dictated by epitope binding emerge as more critical parameters than mere binding affinity. Furthermore, the superior tumor uptake achieved with the pertuzumab-TCO strategy is likely collectively contributed to by its higher TCO payload, lower tumor internalization rate, and slower systemic clearance. Notably, in preliminary studies, trastuzumab-TCO uptake dropped to nearly undetectable levels by day 7, necessitating a focus on earlier imaging windows. Although this precludes a fully matched comparison with pertuzumab-TCO, the existing data robustly delineate the distinct pharmacokinetic profiles of the two conjugates.

To empirically validate the advantage of the pretargeting strategy, we directly compared the PET imaging data of the directly labeled antibody probe ([^18^F]TzTCO-trastuzumab/pertuzumab) with our pretargeted system. Due to the relatively short biological half-life of ^18^F, tracking in vitro-labeled antibodies is inherently restricted to a few hours. The comparative data demonstrated that the imaging contrast of the pretargeted trastuzumab/pertuzumab-TCO combined with [^18^F]PEG_12_-Tz system was significantly superior to the imaging results obtained 8 h after the direct injection of [^18^F]TzTCO-trastuzumab/pertuzumab. This comparison confirms that pretargeting effectively overcomes the kinetic mismatch between slow-clearing antibodies and short-lived isotopes, providing a robust solution for high-contrast, same-day immuno-PET imaging.

Our findings establish a practical, mechanism-informed framework for antibody selection and engineering in pretargeted applications. For clinical translation, the choice may be adapted to specific requirements: pertuzumab-based pretargeting offers superior performance for applications necessitating high signal intensity and an extended imaging window, such as quantitative lesion assessment or when scheduling flexibility is desired. Conversely, the faster clearance profile of trastuzumab-based pretargeting, despite its shorter window, may be advantageous in scenarios requiring rapid turnaround. More broadly, this study suggests that future antibody engineering for pretargeting should emphasize designing molecules or fragments with intrinsically low internalization rates to optimize the efficiency of the bioorthogonal click reaction at the target site.

We acknowledge several limitations of the current study. First, the unequal TCO-to-antibody ratios (30 vs. 13) between the two conjugates may confound the separation of DAR effects from epitope-specific biological mechanisms. Although the TCO loading was characterized, its precise influence on pharmacokinetics and imaging contrast requires further systematic assessment. Future investigations should therefore evaluate a series of matched TCO loadings for both antibodies to decouple these contributions. Second, this study lacks direct quantification of reactive TCO groups. The actual number of functionally available TCO tags for click chemistry may differ from the total TCO loading due to isomerization or steric hindrance. Future work should employ fluorogenic tetrazine probes to directly validate functional TCO availability. Third, the evaluation was conducted exclusively in a single HER2-positive xenograft model (SKOV3). Subsequent studies should validate these observations in additional models with varying HER2 expression levels and in metastatic contexts to determine broader applicability. Finally, comprehensive radiopharmaceutical profiling—such as plasma protein binding and in vivo metabolite analysis—was not performed. While beyond the scope of this proof-of-concept study, such data are essential for future clinical translation and should be addressed in subsequent investigations.

## 4. Materials and Methods

### 4.1. Reagents and Instruments

All reagents were commercially obtained and used as received unless otherwise specified. Trastuzumab and pertuzumab were sourced from Roche Diagnostics GmbH (Sandhofer Strasse 116, Mannheim, Germany). TCO-PEG_4_-NHS Ester and NOTA-PEG_12_-Tz were purchased from Xi’an Ruixi Biological Technology Co., Ltd. (Xi’an, Shaanxi, China). and Nanchang Tanzhen Biotechnology Co., Ltd. (Nanchang, Jiangxi, China), respectively. [^18^F]Fluoride was produced as an [^18^O]-enriched aqueous solution using a Sumitomo HM-20 cyclotron (Dongcheng AMS Pharmaceutical (Dongguan, Guangdong, China). Solid-phase extraction was performed using Sep-Pak QMA Light and C18 Light cartridges (Waters, Milford, MA, USA). Radioactivity quantification was carried out using a Capintec CAPRAC-R dose calibrator (Capintec, Inc., Florham Park, NJ, USA) and a Hidex γ-counter (Hidex Oy, Turku, Finland), while analytical characterization was achieved on an Agilent 1260 Infinity II HPLC system (Agilent Technologies, Inc., Santa Clara, CA, USA). Furthermore, in vivo imaging data were acquired using a Madiclab PSA146 PET/CT/FMT scanner (Shandong Madic Health Technology Co., Ltd., Linyi, Shandong, China).

### 4.2. Conjugation and Purification of Trastuzumab/Pertuzumab-TCO

Initial buffer exchange and purification of the humanized HER2 antibodies (trastuzumab and pertuzumab) were achieved using a pre-equilibrated PD-10 desalting column. In preparation for TCO conjugation, the pH of the antibody solution (300 µL, 20–30 mg/mL in PBS) was adjusted to 8.5–9.0 by introducing 9 µL of a saturated K_2_CO_3_ solution. Subsequently, 20 µL of TCO-PEG_4_-NHS ester in DMSO (50 mg/mL) was added, and the reaction mixture was stirred at room temperature for 60 min. The product was purified using a pre-equilibrated PD-10 column (PBS, pH 7.4). The resulting antibody-TCO conjugates (trastuzumab-TCO and pertuzumab-TCO) were stored in PBS at 4 °C. Conjugate purity and protein concentration were determined by size-exclusion HPLC (BioCoreTM SEC column, PBS, 1 mL/min). The average number of TCO moieties per antibody (drug-to-antibody ratio, DAR) was calculated by MALDI-TOF-MS based on the mass shift relative to the unconjugated antibody.

### 4.3. Radiosynthesis of [^18^F]PEG_12_-Tz

[^18^F]Fluoride was initially retained on a preconditioned QMA cartridge (equilibrated with 5 mL of 0.5 M NaOAc buffer, pH 3.9, followed by 5 mL of water) and subsequently recovered by elution with 0.3 mL of the same NaOAc buffer. The resulting eluate (300 µL, 2–5 GBq) was combined with AlCl_3_ (10 µL, 2 mM), DMSO (300 µL), and NOTA-PEG_12_-Tz (50 µL, 4 mM). The reaction mixture was then incubated at 100 °C for 15 min. Following thermal treatment, the solution was chilled in an ice bath, diluted with 9 mL of water, and passed through a preconditioned C18 cartridge. After rinsing the cartridge with 30 mL of water, the desired product was isolated by elution with 1.5 mL of a 1:1 (*v*/*v*) ethanol/water solution, which was then subjected to sterile filtration (0.2 µm). Radiochemical purity was assessed by radio-HPLC using an analytical ChromCore C18 column (5 µm, 4.6 × 150 mm, NanoChrom Technologies Co., Ltd., Suzhou, Jiangsu, China) with a gradient of 0.1% TFA in water (A) and 0.1% TFA in acetonitrile (B) at 1 mL/min (90% A to 10% A over 10 min). After C18 cartridge purification and sterile filtration, the molar activity was computed from the ratio of total radioactivity (measured by a dose calibrator) to total mass (determined via analytical HPLC-UV calibration curves).

### 4.4. Partition Coefficient

The octanol-water distribution coefficient (LogD) was assessed using a modified shake-flask method. Briefly, [^18^F]PEG_12_-Tz (37 kBq) was introduced into a two-phase system comprising 5.0 mL of PBS (pH 7.4) and 5.0 mL of 1-octanol within a 15 mL centrifuge tube. Following intensive vortex mixing for 5 min and high-speed centrifugation at 10,000 rpm for 5 min, distinct phase separation was achieved. Precise 100 µL aliquots were then extracted from both the upper (octanol) and lower (PBS) phases, with radioactivity quantified on a γ-counter. The final LogD value was computed as Log_10_ (octanol counts/PBS counts) (n = 3).

### 4.5. In Vitro Ligation of [^18^F]PEG_12_-Tz with Trastuzumab/Pertuzumab-TCO

The radioligand [^18^F]PEG_12_-Tz (500 µL, 0.37 GBq) was combined with the corresponding TCO-conjugated antibody (300 µg, ~2 nmol), followed by co-incubation under gentle agitation for 10 min at room temperature. After in vitro ligation of [^18^F]PEG_12_-Tz with antibody-TCO conjugates (10 min, RT), the reaction mixture was purified via PD-10 desalting columns (GE Healthcare) equilibrated with PBS (pH 7.4). The conjugated product ([^18^F]TzTCO-trastuzumab/pertuzumab) was eluted in the void volume (3–5 mL), while free [^18^F]PEG_12_Tz (MW ~1.2 kDa) was retained in the column matrix and eluted later (8–10 mL). Post-purification, radiochemical purity of antibody conjugates ([^18^F]TzTCO-trastuzumab/pertuzumab) was analyzed by size-exclusion HPLC (SEC-HPLC).

### 4.6. In Vitro Stability

#### 4.6.1. Storage Stability

Pertuzumab-TCO conjugate was stored in PBS (pH 7.4) at 4 °C for 7 days, and its stability was subsequently evaluated by SEC-HPLC.

#### 4.6.2. Biological Stability

[^18^F]PEG_12_-Tz and [^18^F]TzTCO-pertuzumab were spiked into normal human serum (0.2 mL) and incubated at 37 °C for 1 h. After precipitation with 0.4 mL acetonitrile and centrifugation (10,000 rpm, 5 min), the supernatant was analyzed by analytical Radio-C18-HPLC and Radio-SEC-HPLC, respectively, to determine radiochemical purity.

### 4.7. Cell Line and Tumor Model

The SKOV3 (HER2-high) and MDA-MB-231 (HER2-low) cell lines, procured from the Institute of Biochemistry and Cell Biology (Shanghai, China), served as the in vitro model systems. These cells were maintained in RPMI-1640 or DMEM medium, respectively, fortified with 15% fetal bovine serum and 1% penicillin-streptomycin (all from Gibco, Grand Island, NY, USA), and incubated at 37 °C in a humidified atmosphere containing 5% CO_2_.

For in vivo experiments, female BALB/c-Nude mice (4–6 weeks of age, 13–18 g) were subcutaneously implanted with 5 × 10^6^ cells (in 100 µL PBS) into the right flank. Subsequent imaging and biodistribution studies were commenced once the resulting tumor xenografts attained a diameter of 5–10 mm. Healthy female BALB/cJGpt mice (4–6 weeks of age, 13–18 g) were recruited for the biodistribution and pharmacokinetic evaluations.

### 4.8. Cell Assay

#### 4.8.1. Western Blot Analysis

Western blotting was performed to assess HER2 expression levels in SKOV3 and MDA-MB-231 cells, as detailed in the Supplementary Materials.

#### 4.8.2. In Vitro Pre-Targeting Specificity

The specific pretargeting capability of trastuzumab-TCO and pertuzumab-TCO was evaluated in HER2-high SKOV3 cells. Cells were seeded and divided into four experimental groups. In the pretargeting group, cells were first incubated with the respective antibody-TCO conjugate (100 µg) for 1 h at 37 °C, washed twice with cold PBS (pH = 7.4), and subsequently incubated with [^18^F]PEG_12_-Tz (74 kBq) for an additional hour. Following removal of the medium and two further washes, cells were lysed with 1 N NaOH. The radioactivity in the lysate was measured using a γ-counter and expressed as the percentage of the injected dose per 10^6^ cells (%ID/10^6^ cells). Two blocking experiments were also performed. In these experiments, cells were pretreated with excessive unmodified trastuzumab or pertuzumab (5 mg) for 1 h at 37 °C before TCO-conjugate addition. To compete for tetrazine binding, 50 μg/mL unlabeled NOTA-PEG_12_-Tz was present during the 1 h radioligand uptake assay. As an additional control, [^18^F]PEG_12_-Tz was incubated with cells without prior antibody-TCO treatment. All groups underwent identical washing, lysis, and measurement procedures.

#### 4.8.3. Cellular Uptake, Internalization and Efflux Experiments of [^18^F]TzTCO-Trastuzumab/Pertuzumab

For cellular uptake studies, SKOV3 cells were exposed to the radiolabeled compound for 5, 30, 60, and 120 min at 37 °C. The incubation was terminated by aspirating the medium and rinsing the cells twice with 1 mL of ice-cold PBS. Internalization assays were conducted by treating SKOV3 cells with [^18^F]TzTCO-Trastuzumab or [^18^F]TzTCO-Pertuzumab for 60 min at 37 °C. Following medium removal and two washes with ice-cold PBS, membrane-bound radioactivity was stripped by incubating the cells with 1 M glycine-HCl (pH 2.2) for 10 min at 37 °C. After further washing, cells were lysed, and the radioactivity in the lysate was quantified to determine the internalized fraction. Efflux kinetics were evaluated by replacing the radiolabeled incubation medium with fresh, nonradioactive medium after an initial 60 min incubation, followed by continued culture for various durations (0–180 min) to monitor tracer release.

#### 4.8.4. Saturation Binding Experiments

Increasing concentrations (0.08–10 nM) of purified radioligand ([^18^F]TzTCO-Trastuzumab/Pertuzumab) were incubated with SKOV3 cells (2 × 10^5^ cells/well) for 1 h at 37 °C. Nonspecific binding was determined in parallel wells pre-incubated with a 100-fold molar excess of the corresponding unlabeled antibody. After incubation, cells were washed, lysed, and the bound radioactivity was quantified. Each concentration point was assayed in triplicate. The equilibrium dissociation constant (Kd) was extrapolated through nonlinear curve fitting based on a one-site binding paradigm, executed within the GraphPad Prism 9.5 software environment.

### 4.9. Micro-PET/CT Imaging and Immunohistochemical Staining

To determine the optimal pretargeting time window and compare the in vivo pharmacokinetics of the two antibody conjugates, pretargeted PET imaging was performed. Female BALB/c-Nude mice bearing SKOV3 xenografts were intravenously injected with 300 µg of trastuzumab/pertuzumab-TCO. For the pretargeted imaging cohorts, the radioligand [^18^F]PEG_12_-Tz was subsequently administered intravenously at pretargeted time points. Specifically, for the pertuzumab-TCO group, [^18^F]PEG_12_-Tz (3.7 MBq, 1.16 nM, 1.34 μg) was injected on days 1, 3, 5, 7, 9, 11, 13, and 15 with post-antibody injection. For the trastuzumab-TCO group, due to its more rapid clearance and shorter imaging window, the radioligand was administered on days 1, 3, 5, and 7. Static micro-PET/CT scans were acquired 1 h after radioligand administration. After identifying the optimal time point, the same pretargeting protocol was applied to mice bearing HER2-low MDA-MB-231 xenografts as a control group. Specificity was further validated at the optimal time point through blocking experiments: (1) co-injection of a 50-fold molar excess of the corresponding unlabeled antibody (15 mg) with the TCO conjugate on Day 0, and (2) Co-administration of an excess of unlabeled NOTA-PEG_12_-Tz (50 μg) alongside [^18^F]PEG_12_-Tz (trastuzumab-TCO on Day 5; pertuzumab-TCO on Day 9). Direct PET imaging was performed by injecting tumor-bearing mice with [^18^F]TzTCO-Trastuzumab/Pertuzumab (7.4 MBq/mouse) via the tail vein.

Small-animal PET/CT imaging was performed under isoflurane anesthesia (2–3% in 1 L/min O_2_) using a Madiclab PSA146 PET/CT/FMT system. Body temperature was regulated using a heating pad during imaging. The raw PET data were reconstructed via a 3D OSEM algorithm. Subsequently, ROI-based quantification was performed on decay-corrected coronal images using PMOD (v4.3, PMOD Technologies Ltd., Zurich, Switzerland) to determine tissue uptake, which was standardized to %ID/g for both neoplastic and healthy tissues. The optimal imaging window was defined as the interval during which (1) tumor uptake exceeded 1.0% ID/g, and (2) tumor-to-muscle ratio (TMR) was ≥5.0.

Histological evaluation via immunohistochemistry (IHC) was conducted on 3-μm-thick tissue sections derived from SKOV3 and MDA-MB-231 tumor xenografts. Comprehensive experimental details are elaborated in the Supplementary Materials.

### 4.10. Biodistribution

Female BALB/c-Nude mice bearing SKOV3 xenografts were intravenously injected via the tail vein with trastuzumab-TCO or pertuzumab-TCO (300 μg, 150 μL PBS). Mice receiving trastuzumab-TCO were administered [^18^F]PEG_12_-Tz (1.85 MBq, 0.58 nM, 0.67 μg) 5 days later, whereas those receiving pertuzumab-TCO were injected with the radioligand after 9 days. The negative control experiment was conducted in mice bearing HER2-low MDA-MB-231 xenografts following the same protocol. For blocking experiments, mice were co-injected with excessive trastuzumab or pertuzumab (15 mg) together with the corresponding TCO conjugate on Day 0, and/or 50 μg of unlabeled NOTA-PEG_12_-Tz together with [^18^F]PEG_12_-Tz (Day 5 for trastuzumab-TCO; Day 9 for pertuzumab-TCO) to block the binding of the antibody-TCO or the radioligand, respectively.

To assess the pharmacokinetics and systemic exposure, female normal mice (BALB/cJGpt) received a single intravenous dose of trastuzumab-TCO or pertuzumab-TCO (300 μg/mouse). At specified time points (1, 24, 72, and 120 h; n = 3 mice per group), [^18^F]PEG_12_-Tz (1.85 MBq) was administered. Mice were sacrificed 1 h later for the collection of blood and major organs. Uptake was quantified as %ID/g, and non-compartmental analysis via GraphPad Prism was used to determine the terminal half-life (t_1/2β_).

### 4.11. Statistical Analysis

Quantitative data are presented as means ± standard deviation (SD). All pairwise comparisons use the Mann–Whitney U test, and multigroup comparisons use the Kruskal–Wallis test with Dunn’s post hoc correction. All statistical analyses were performed using GraphPad Prism version 9.5 (GraphPad Software, San Diego, CA, USA). A *p*-value < 0.05 was considered statistically significant.

## 5. Conclusions

In summary, this study demonstrates that antibody selection is a critical determinant of performance in bioorthogonal pretargeted PET. Direct platform comparisons demonstrate the distinct superiority of pertuzumab-TCO over trastuzumab-TCO, driven by its higher TCO loading, reduced tumor internalization, and slower clearance kinetics. This translates into superior tumor uptake, prolonged retention, and an expanded imaging window. These findings underscore that antibodies targeting the same antigen are not functionally interchangeable in multi-step pretargeting. Our work provides a practical, mechanism-based framework for selecting or engineering antibodies to optimize pretargeted imaging protocols.

## Data Availability

The original contributions presented in the study are included in the article and Supplementary Material, further inquiries can be directed to the corresponding author.

## Supplementary Materials

The following supporting information can be downloaded at: https://www.mdpi.com/article/10.3390/ph19081276/s1, Figure S1: The MALDI-TOF-MS of trastuzumab; Figure S2: The MALDI-TOF-MS of pertuzumab; Figure S3: The MALDI-TOF-MS of trastuzumab-TCO; Figure S4: The MALDI-TOF-MS of pertuzumab-TCO; Figure S5: The SEC-HPLC of trastuzumab-TCO; Figure S6: The SEC-HPLC of pertuzumab-TCO; Figure S7: The quality control radioactive chromatography of [^18^F]PEG_12_-Tz (red line: the radioactive profile; black line: UV profile); Figure S8: In vitro Stability of pertuzumab-TCO; Figure S9: In vitro Stability of [^18^F]PEG_12_-Tz; Figure S10: In vitro Stability of [^18^F]TzTCO-Pertuzumab; Figure S11: Protein expression of HER2 in SKOV3 and MDA-MB-231 cell lines was analyzed by Western blotting. Figure S12. Efflux kinetics of [^18^F]TzTCO-Trastuzumab and [^18^F]TzTCO-Pertuzumab after 60 min incubation of SKOV3 cells with tracers followed by incubation with compound-free medium for 0–180 min. Figure S13. Saturation binding curve of [^18^F]TzTCO-Trastuzumab and [^18^F]TzTCO-Pertuzumab. Figure S14. (A) Micro-PET images of pre-injected with trastuzumab-TCO (300 μg in PBS) followed 1 d, 3 d, 5 d, and 7 d later by [^18^F]PEG_12_-Tz, where white arrow circle indicates tumor area. (B) Uptake value of SKOV3 tumor at 1 h post-injection of [^18^F]PEG_12_-Tz. (C) The ratios of SKOV3 tumor/heart, tumor/muscle and tumor/liver. Table S1. Biodistribution of female BALB/c nude mice bearing subcutaneous SKOV3/MDA-MB-231 xenografts pre-injected with trastuzumab-TCO (300 μg in PBS) followed 120 h later by [^18^F]PEG_12_-Tz. The animals were euthanized, and tissues of interest were harvested at 1 h after [^18^F]PEG_12_-Tz injection. Table S2. Biodistribution of female BALB/c nude mice bearing subcutaneous SKOV3/MDA-MB-231 xenografts pre-injected with pertuzumab-TCO (300 μg in PBS) followed 216 h later by [^18^F]PEG_12_-Tz. The animals were euthanized, and tissues of interest were harvested at 1 h after [^18^F]PEG_12_-Tz injection. Table S3. Biodistribution of female normal mice (BALB/cJGpt) pre-injected with trastuzumab-TCO (300 μg in PBS) followed 1, 24, 72 and 120 h later by [^18^F]PEG_12_-Tz. The animals were euthanized, and tissues of interest were harvested at 1 h after [^18^F]PEG_12_-Tz injection. Table S4. Biodistribution of normal mice (BALB/cJGpt) pre-injected with pertuzumab-TCO (300 μg in PBS) followed 1, 24, 72 and 120 h later by [^18^F]PEG_12_-Tz. The animals were euthanized, and tissues of interest were harvested at 1 h after [^18^F]PEG_12_-Tz injection. Figure S15. Static PET images obtained 1, 4, and 8 h after direct injection of [^18^F]TzTCO-Trastuzumab (A) and [^18^F]TzTCO-Pertuzumab (B) in male mice with subcutaneous implanted SKOV3 xenograft tumors. Figure S16. The ratios of SKOV3 tumor/muscle (A) and tumor/liver (B). Figure S17. The pharmacokinetic curve of trastuzumab-TCO in blood. Figure S18. The pharmacokinetic curve of pertuzumab-TCO in blood.

## Abbreviations

The following abbreviations are used in this manuscript:

PET	Positron emission tomography
IEDDA	Inverse Electron Demand Diels-Alder reaction
TCO	Trans-cyclooctene
HER2	Human Epidermal Growth Factor Receptor 2
mAb	Monoclonal Antibody
SEC	Size Exclusion Chromatography
NHS	N-Hydroxysuccinimide
PEG	Polyethylene glycol
NOTA	1,4,7-triazacyclononane-N,N′,N″-triacetic acid
DMSO	Dimethyl sulfoxide
NaOAc	Sodium acetate
K_2_CO_3_	Potassium carbonate
AlCl_3_	Aluminum chloride
PBS	Phosphate-buffered saline
QMA	Quaternary Methyl Ammonium
TFA	Trifluoroacetic acid
HPLC	High performance liquid chromatography
UV	Ultraviolet
MALDI-TOF-MS	Matrix-Assisted Laser Desorption/Ionization Time-of-Flight Mass Spectrometry
GAPDH	Glyceraldehyde-3-phosphate dehydrogenase
IHC	Immunohistochemistry
TMR	Tumor muscle ratio
MIP	Maximum density projection
OSEM	Ordered-subset expectation maximization
SD	Standard Deviation
DAR	Drug-to-Antibody Ratio
