# Supplementary material for: A Head-to-Head Comparison of Two Antibodies for HER2 Pretargeted PET Imaging via Bioorthogonal Click Chemistry
Source: Pharmaceuticals (Basel). 2026 Aug 13;19(8):1276. doi: 10.3390/ph19081276 (PMC13516066; doi:10.3390/ph19081276)
Supplement: Supplementary file 1 [file pharmaceuticals-19-01276-s001.zip › pharmaceuticals-4372571-supplementary.pdf]

Supplemental Information

A Head-to-Head Comparison of Two Antibodies for HER2 Pretargeted PET Imaging via Bioorthogonal Click Chemistry

Yong Huang †, Chengze Li †, Taichuang Li, Jiuhui Zhao, Xinyu Yang, Maoqun Zhang and Ying Liang *

Department of Nuclear Medicine, National Cancer Center/National Clinical Research Center for Cancer/Cancer Hospital & Shenzhen Hospital, Chinese Academy of Medical Sciences and Peking Union Medical College, Shenzhen 518116, China; 13260455651@163.com (Y.H.); lichengze1915@163.com (C.L.); 17688426991@139.com (T.L.); izljkh@163.com (J.Z.); 13875015513@163.com (X.Y.); libseveny@163.com (M.Z.)

* Correspondence: liangying@cicams-sz.org.cn

† These authors contributed equally to this work.

Content

1. Characterization S2

2. Stability S4

3. Western Blot S5

4. Cellular Efflux S6

5. Immunohistochemical Staining S6

6. Saturation binding. S7

7. PET imaging and biodistribution. S7

8. The pharmacokinetic curve. S12

1. Characterization

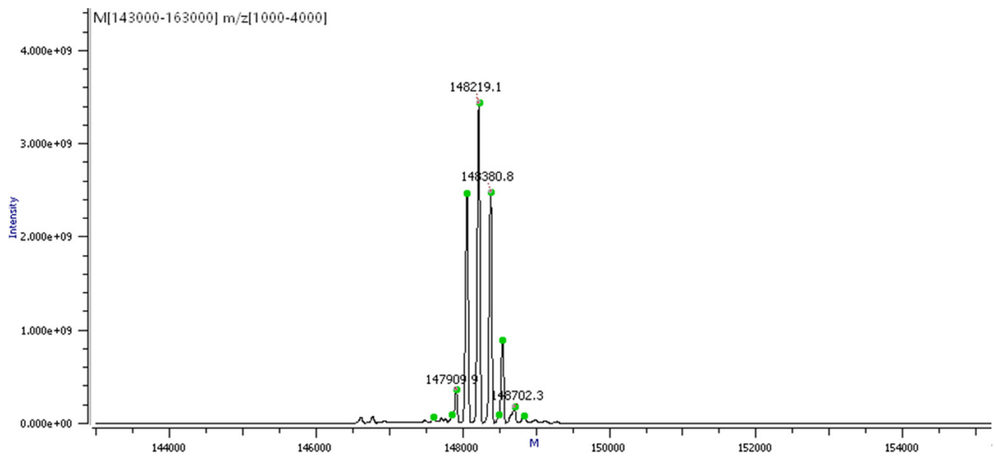

Figure S1. The MALDI-TOF-MS of trastuzumab

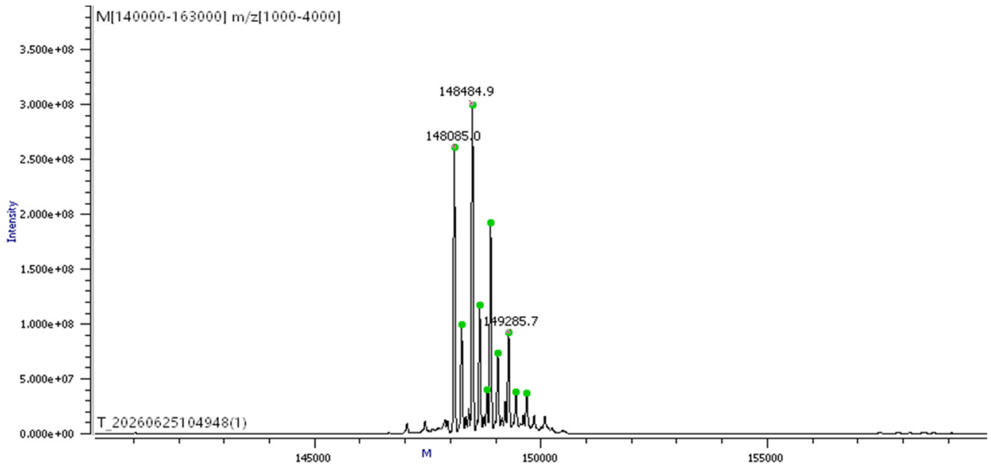

Figure S2. The MALDI-TOF-MS of pertuzumab

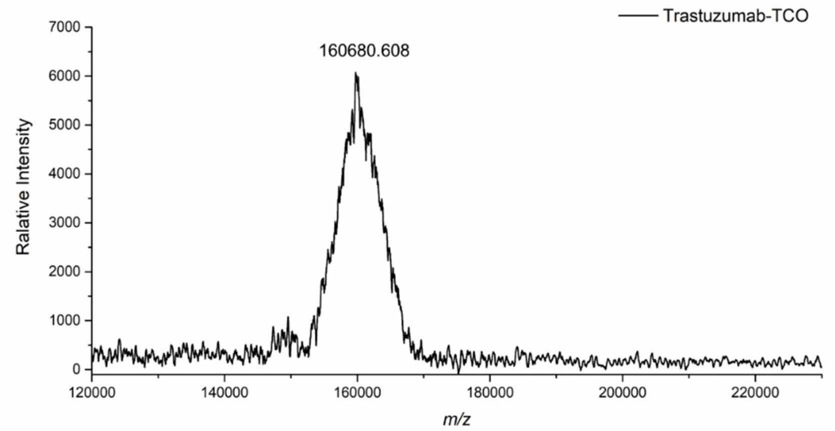

Figure S3. The MALDI-TOF-MS of trastuzumab-TCO

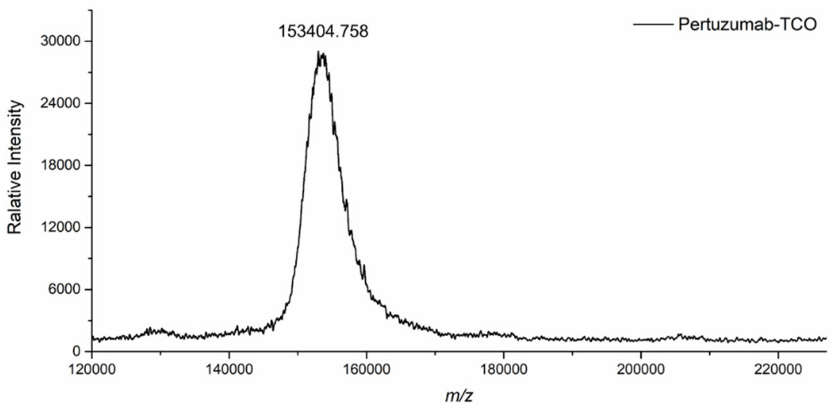

Figure S4. The MALDI-TOF-MS of pertuzumab-TCO

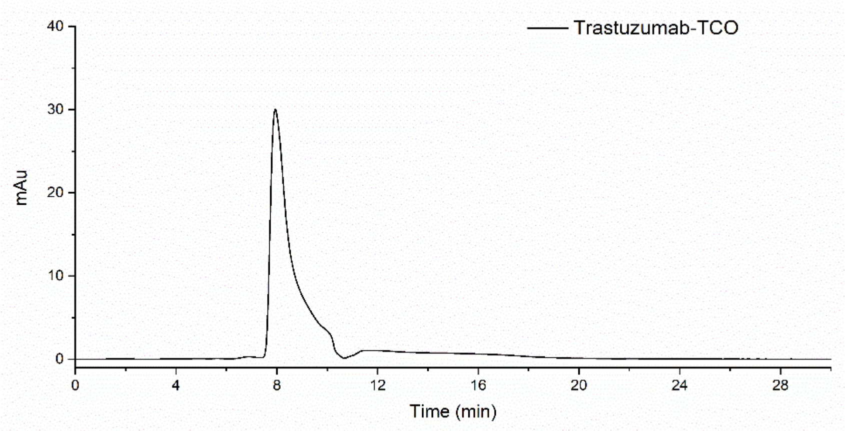

Figure S5. The SEC-HPLC of trastuzumab-TCO

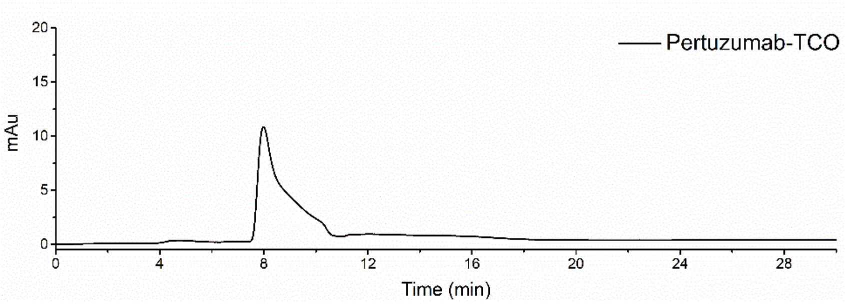

Figure S6. The SEC-HPLC of pertuzumab-TCO

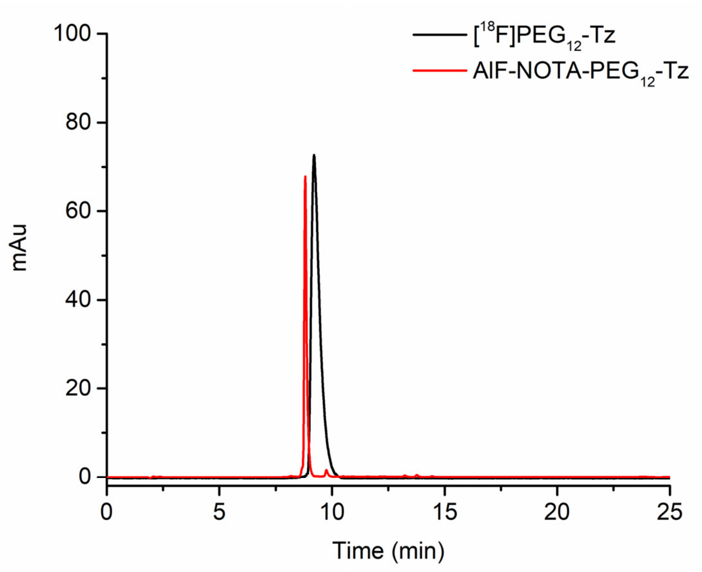

Figure S7. The quality control radioactive chromatography of $[^{18}F]PEG_{12}$-Tz (red line: the radioactive profile; black line: UV profile).

2. Stability

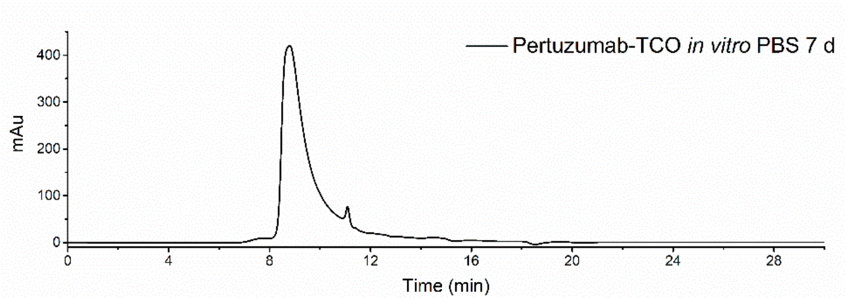

Figure S8. In vitro Stability of pertuzumab-TCO

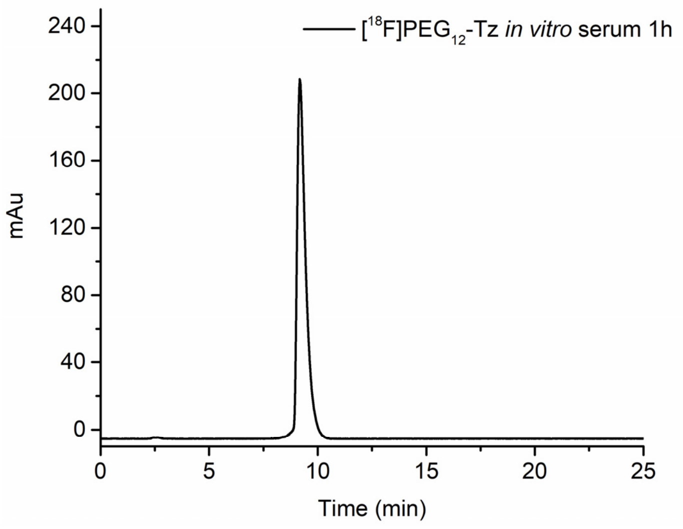

Figure S9. In vitro Stability of $[^{18}F]PEG_{12}$-Tz

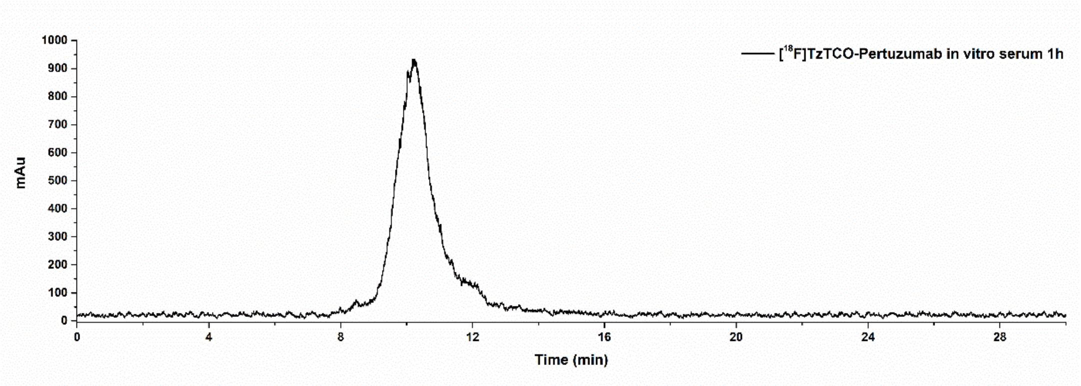

Figure S10. In vitro Stability of [18F]TzTCO-Pertuzumab

3. Western Blot

Protein expression of HER2 in SKOV3 and MDA-MB-231 cell lines was assessed by Western blot. Briefly, cells were lysed using RIPA buffer (Keygen Biotech, Nanjing, China) supplemented with protease and phosphatase inhibitors. Protein concentration was determined with the Bradford protein assay (Keygen Biotech, Nanjing, China). Proteins were then denatured by boiling with loading buffer at 95 °C for 10 min. Total protein (20 μg per lane) was separated by SDS-PAGE using a commercial kit (Dingguo, Nanjing, China) and transferred onto polyvinylidene difluoride membranes. The membranes were blocked with 5% bovine serum albumin for 1 h, followed by overnight incubation at 4 °C with a polyclonal rabbit anti-HER2 primary antibody (Abcam, Cambridge, UK) diluted at 1:800. After washing, membranes were incubated for 2 h with horseradish peroxidase-conjugated anti-rabbit IgG (weiao, Xian, China) at 1:2000 dilution. Protein bands were visualized using enhanced chemiluminescence and imaged with an Alliance MINI HD6 analyzer (UVITEC, UK). Grayscale analysis was performed with UVI-1D software. GAPDH was used as the internal control.

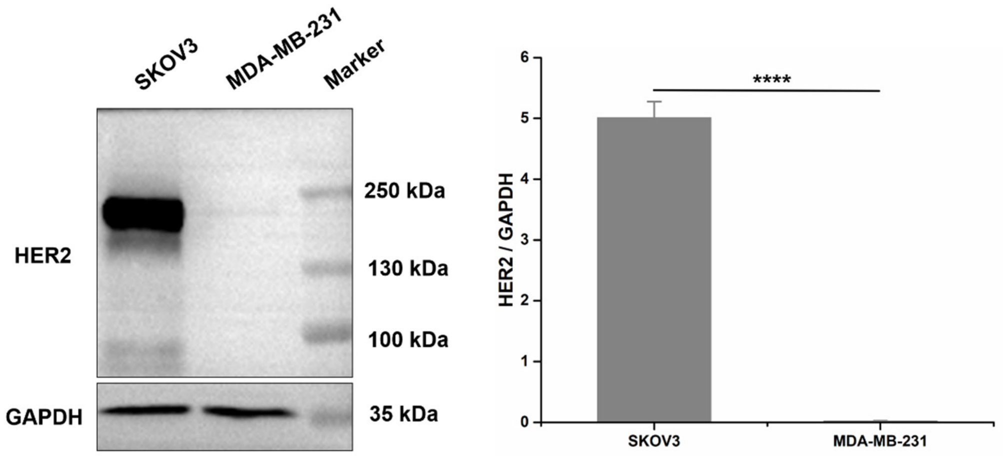

Figure S11. Protein expression of HER2 in SKOV3 and MDA-MB-231 cell lines was analyzed by western blotting. Statistical significance was indicated as ****P < 0.001. Values are expressed as means ± SD (% ID/g) (n = 3).

4. Cellular Efflux

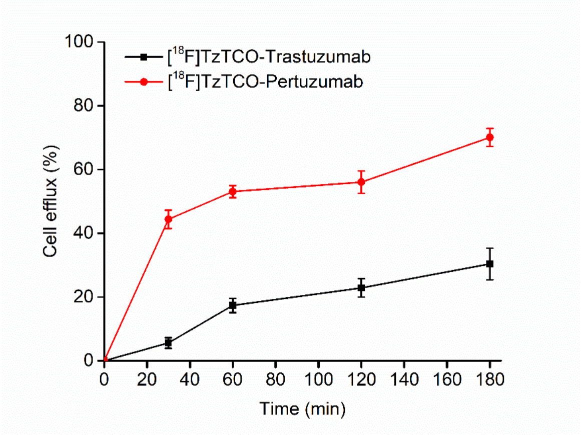

Figure S12. Efflux kinetics of [^{18}F]TzTCO-Trastuzumab and [^{18}F]TzTCO-Pertuzumab after 60 min incubation of SKOV3 cells with tracers followed by incubation with compound-free medium for 0–180 min. Values are expressed as means ± SD (% ID/g) (n = 3).

5. Immunohistochemical Staining

Tumor tissues of SKOV3 and MDA-MB-231 tumor-bearing mice obtained by sacrificing the mice were immunohistochemically stained after preparation of paraffin-embedded sections using 4% paraformaldehyde. The sections were incubated with the HER2-alpha primary antibody (diluted 1:200, Affinity) and then with the goat anti-mouse secondary antibody (diluted 1:200, Servicebio). The secondary antibody was a molecule formed by combining horseradish peroxidase (HRP) and goat anti-mouse IgG.

The sections were then reacted with DAB staining solution after combining the primary and secondary antibodies. DAB produced brown precipitation upon HRP catalysis, which amplified the signal and developed color. The immunohistochemical images were finally obtained after a series of routine processing.

6. Saturation binding

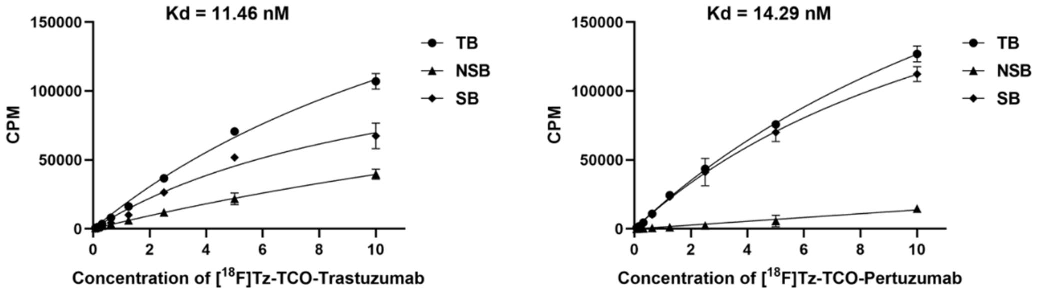

Figure S13. Saturation binding curve of [^{18}F]TzTCO-Trastuzumab and [^{18}F]TzTCO-Pertuzumab. NS: non-specific binding; TB: total binding; SB: specific binding. Values are expressed as means ± SD (% ID/g) (n = 3).

7. PET imaging and biodistribution

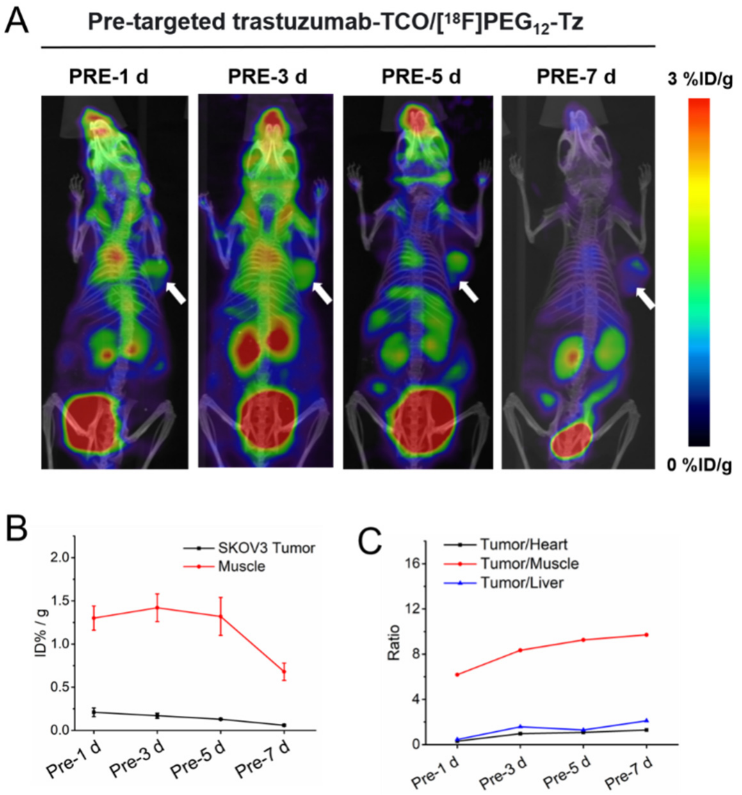

Figure S14. (A) Micro-PET images of pre-injected with trastuzumab-TCO (300 μg in PBS)

followed 1 d, 3 d, 5 d, and 7 d later by [^{18}F]PEG$_{12}$-Tz, where white arrow circle indicates tumor area. (B) Uptake value of SKOV3 tumor at 1 h post-injection of [^{18}F]PEG$_{12}$-Tz. (C) The ratios of SKOV3 tumor/heart, tumor/muscle and tumor/liver. Values are expressed as means ± SD (% ID/g) (n = 3).

Table S1. Biodistribution of female BALB/c nude mice bearing subcutaneous SKOV3/MDA-MB-231 xenografts pre-injected with trastuzumab-TCO (300 μg in PBS) followed 120 h later by [^{18}F]PEG$_{12}$-Tz. The animals were euthanized, and tissues of interest were harvested at 1 h after [^{18}F]PEG$_{12}$-Tz injection. Values are expressed as means ± SD (% ID/g) (n = 3).

Organ	Uptake (SKOV3)	Uptake (MDA-MB-231)	Tz Block (SKOV3)	mAb Block (SKOV3)
Bone	0.16 ± 0.02	0.06 ± 0.01	0.19 ± 0.04	0.18 ± 0.02
Muscle	0.13 ± 0.02	0.12 ± 0.01	0.12 ± 0.02	0.13 ± 0.02
Lung	0.35 ± 0.06	0.31 ± 0.03	0.47 ± 0.02	0.40 ± 0.04
Brain	0.04 ± 0.01	0.01 ± 0.01	0.03 ± 0.01	0.02 ± 0.00
Heart	0.24 ± 0.03	0.11 ± 0.03	0.20 ± 0.03	0.17 ± 0.06
Liver	0.50 ± 0.06	0.26 ± 0.03	0.17 ± 0.05	0.20 ± 0.05
Kidney	1.61 ± 0.17	1.06 ± 0.16	0.83 ± 0.22	1.23 ± 0.22
Spleen	0.28 ± 0.05	0.23 ± 0.05	0.14 ± 0.24	0.12 ± 0.05
Stomach	0.16 ± 0.03	0.13 ± 0.00	0.12 ± 0.05	0.15 ± 0.05
Intestine	0.29 ± 0.05	0.10 ± 0.01	0.32 ± 0.01	0.08 ± 0.04
Blood	0.67 ± 0.06	0.60 ± 0.01	0.36 ± 0.02	0.29 ± 0.05
Tumor	0.55 ± 0.04	0.25 ± 0.06	0.35 ± 0.06	0.28 ± 0.08
Tumor/Muscle	4.23 ± 0.53	2.08 ± 0.35	2.92 ± 0.36	2.15 ± 0.54
Tumor/Blood	0.82 ± 0.23	0.42 ± 0.11	0.97 ± 0.25	0.97 ± 0.34
Tumor/Liver	1.10 ± 0.32	0.96 ± 0.45	2.06 ± 0.55	1.40 ± 0.54

Table S2. Biodistribution of female BALB/c nude mice bearing subcutaneous SKOV3/MDA-MB-231 xenografts pre-injected with pertuzumab-TCO (300 μg in PBS) followed 216 h later by [^{18}F]PEG$_{12}$-Tz. The animals were euthanized, and tissues of interest were harvested at 1 h after [^{18}F]PEG$_{12}$-Tz injection. Values are expressed as means ± SD (% ID/g) (n = 3).

Organ	Uptake (SKOV3)	Uptake (MDA-MB-231)	Tz Block (SKOV3)	mAb Block (SKOV3)
Bone	0.10 ± 0.02	0.07 ± 0.01	0.17 ± 0.04	0.17 ± 0.04
Muscle	0.11 ± 0.03	0.10 ± 0.01	0.13 ± 0.02	0.06 ± 0.03
Lung	0.41 ± 0.04	0.36 ± 0.03	0.47 ± 0.02	0.25 ± 0.03
Brain	0.02 ± 0.01	0.01 ± 0.00	0.04 ± 0.01	0.03 ± 0.01
Heart	0.27 ± 0.03	0.11 ± 0.03	0.27 ± 0.05	0.12 ± 0.04
Liver	0.38 ± 0.05	0.24 ± 0.03	0.17 ± 0.03	0.11 ± 0.02
Kidney	1.62 ± 0.13	1.19 ± 0.16	1.36 ± 0.13	1.20 ± 0.24
Spleen	0.22 ± 0.02	0.15 ± 0.02	0.21 ± 0.02	0.09 ± 0.04
Stomach	0.18 ± 0.04	0.08 ± 0.01	0.05 ± 0.01	0.05 ± 0.01
Intestine	0.36 ± 0.05	0.12 ± 0.01	0.29 ± 0.01	0.29 ± 0.03
Blood	0.63 ± 0.04	0.62 ± 0.05	0.36 ± 0.02	0.25 ± 0.02
Tumor	0.79 ± 0.04	0.24 ± 0.07	0.36 ± 0.05	0.33 ± 0.04
Tumor/Muscle	7.18 ± 0.68	2.40 ± 0.25	2.77 ± 0.56	5.50 ± 0.84
Tumor/Blood	1.25 ± 0.21	0.39 ± 0.15	1.00 ± 0.35	1.32 ± 0.24
Tumor/Liver	2.08 ± 0.42	1.00 ± 0.35	2.12 ± 0.25	3.00 ± 0.64

Table S3. Biodistribution of female normal mice (BALB/cJGpt) pre-injected with trastuzumab-TCO (300 μg in PBS) followed 1, 24, 72 and 120 h later by [^{18}F]PEG$_{12}$-Tz. The animals were euthanized, and tissues of interest were harvested at 1 h after [^{18}F]PEG$_{12}$-Tz injection. Values are expressed as means ± SD (% ID/g) (n = 3).

Organ	Pre-1 h	Pre-24 h	Pre-72 h	Pre-120 h
Bone	1.01 ± 0.17	1.03 ± 0.35	0.74 ± 0.15	0.26 ± 0.04
Muscle	0.58 ± 0.06	0.78 ± 0.23	0.86 ± 0.06	0.42 ± 0.05
Lung	5.79 ± 0.91	7.94 ± 1.25	3.97 ± 0.69	2.06 ± 1.56
Brain	0.21 ± 0.04	0.23 ± 0.05	0.17 ± 0.04	0.09 ± 0.02
Heart	3.59 ± 0.68	3.79 ± 1.23	3.66 ± 0.47	1.47 ± 0.46
Liver	1.79 ± 0.04	2.00 ± 0.52	1.61 ± 0.22	0.83 ± 0.20
Kidney	3.95 ± 0.69	3.13 ± 0.27	2.59 ± 0.24	2.02 ± 0.10
Spleen	0.71 ± 0.04	1.95 ± 0.32	1.54 ± 0.18	0.74 ± 0.10
Stomach	1.70 ± 0.17	0.64 ± 0.26	0.40 ± 0.04	0.17 ± 0.07
Intestine	2.12 ± 0.61	0.95 ± 0.35	0.73 ± 0.15	0.36 ± 0.04

Blood	10.87 ± 0.45	11.85 ± 2.33	9.93 ± 1.46	4.71 ± 1.35

Table S4. Biodistribution of normal mice (BALB/cJGpt) pre-injected with pertuzumab-TCO (300 μg in PBS) followed 1, 24, 72 and 120 h later by $[^{18}F]PEG_{12}$-Tz. The animals were euthanized, and tissues of interest were harvested at 1 h after $[^{18}F]PEG_{12}$-Tz injection. Values are expressed as means ± SD (% ID/g) (n = 3).

Organ	Pre-1 h	Pre-24 h	Pre-72 h	Pre-120 h
Bone	1.15 ± 0.26	0.83 ± 0.08	0.79 ± 0.10	0.42 ± 0.04
Muscle	0.46 ± 0.08	0.67 ± 0.05	0.62 ± 0.01	0.53 ± 0.07
Lung	3.64 ± 0.86	4.81 ± 0.52	4.57 ± 0.39	2.28 ± 0.19
Brain	0.26 ± 0.09	0.20 ± 0.03	0.18 ± 0.03	0.10 ± 0.00
Heart	4.03 ± 0.65	2.99 ± 0.41	2.10 ± 0.32	1.96 ± 0.23
Liver	1.97 ± 0.21	1.57 ± 0.05	1.44 ± 0.15	0.95 ± 0.10
Kidney	3.68 ± 0.54	2.84 ± 0.20	2.57 ± 0.15	2.42 ± 0.03
Spleen	1.57 ± 0.15	1.24 ± 0.12	0.88 ± 0.55	0.72 ± 0.09
Stomach	0.84 ± 0.20	0.92 ± 0.19	0.70 ± 0.04	0.39 ± 0.01
Intestine	1.11 ± 0.55	1.22 ± 0.26	0.88 ± 0.08	0.48 ± 0.26
Blood	12.74 ± 1.42	9.42 ± 0.80	9.36 ± 1.17	5.98 ± 0.57

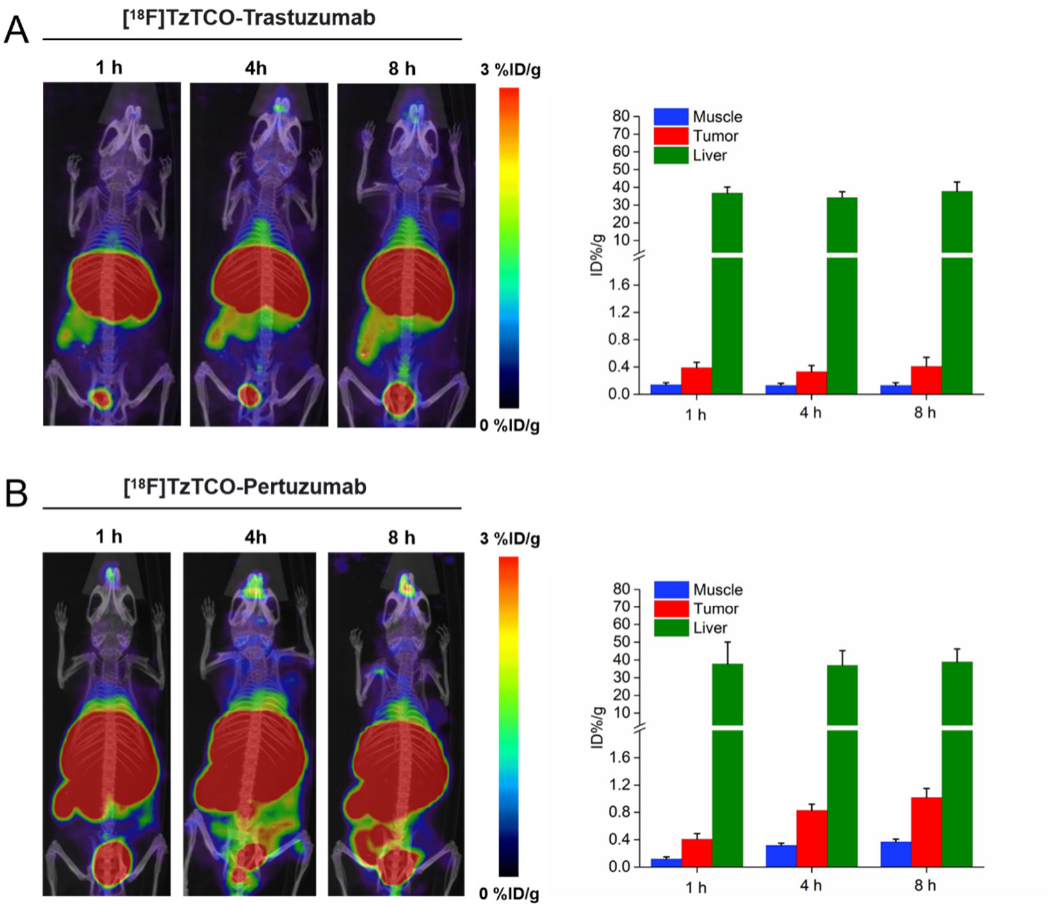

Figure S15. Static PET images obtained 1, 4, and 8 hours after direct injection of [^{18}F]TzTCO-Trastuzumab (A) and [^{18}F]TzTCO-Pertuzumab (B) in male mice with subcutaneous implanted SKOV3 xenograft tumors. Values are expressed as means ± SD (% ID/g) (n = 3).

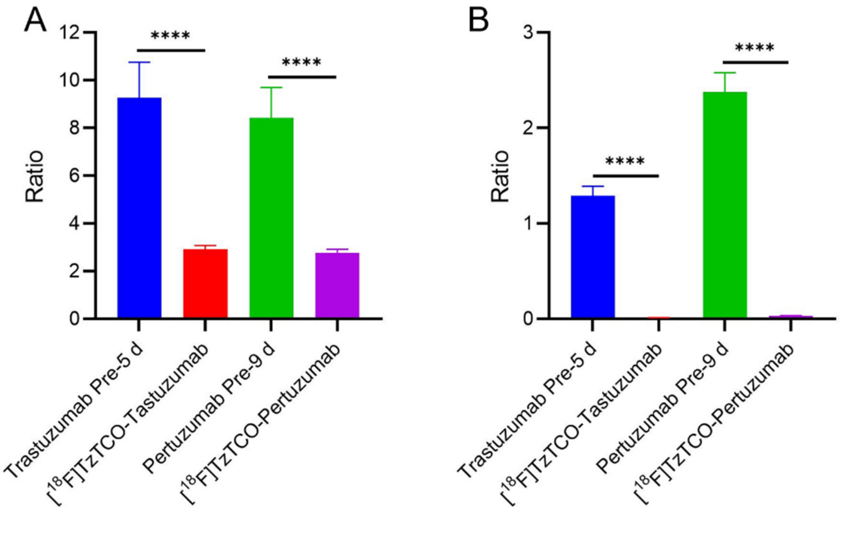

Figure S16. The ratios of SKOV3 tumor/muscle (A) and tumor/liver (B). Statistical

significance was indicated as ****P < 0.001. Values are expressed as means ± SD (% ID/g) (n = 3).

8. The pharmacokinetic curve

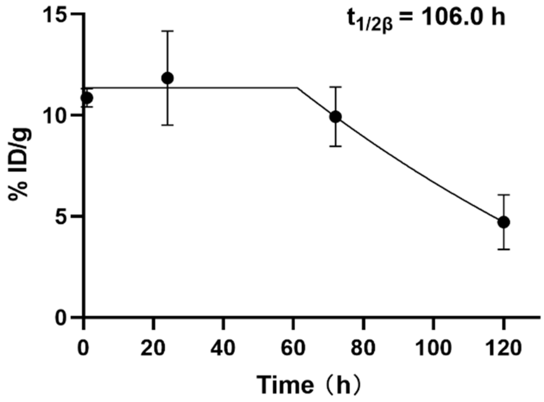

Figure S17. The pharmacokinetic curve of trastuzumab-TCO in blood (n = 3). Values are expressed as means ± SD (% ID/g) (n = 3).

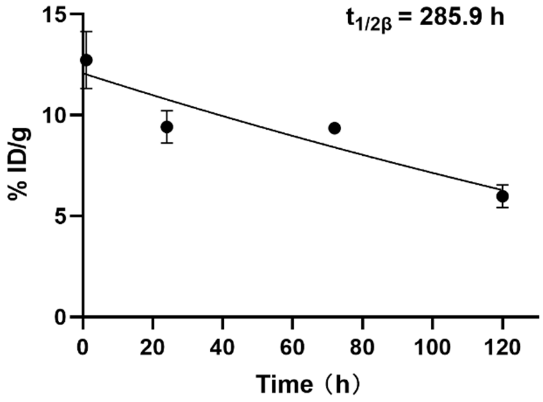

Figure S18. The pharmacokinetic curve of pertuzumab-TCO in blood (n = 3). Values are expressed as means ± SD (% ID/g).
